# A Newton Cooperative Genetic Algorithm Method for *In Silico* Optimization of Metabolic Pathway Production

**DOI:** 10.1371/journal.pone.0126199

**Published:** 2015-05-11

**Authors:** Mohd Arfian Ismail, Safaai Deris, Mohd Saberi Mohamad, Afnizanfaizal Abdullah

**Affiliations:** 1 Faculty of Computer Systems and Software Engineering, Universiti Malaysia Pahang, Lebuhraya Tun Razak, Gambang, Kuantan, Malaysia; 2 Artificial Intelligence and Bioinformatics Group, Department of Software Engineering, Faculty of Computing, Universiti Teknologi Malaysia, Johor, Malaysia; Tel Aviv University, ISRAEL

## Abstract

This paper presents an *in silico* optimization method of metabolic pathway production. The metabolic pathway can be represented by a mathematical model known as the generalized mass action model, which leads to a complex nonlinear equations system. The optimization process becomes difficult when steady state and the constraints of the components in the metabolic pathway are involved. To deal with this situation, this paper presents an *in silico* optimization method, namely the Newton Cooperative Genetic Algorithm (NCGA). The NCGA used Newton method in dealing with the metabolic pathway, and then integrated genetic algorithm and cooperative co-evolutionary algorithm. The proposed method was experimentally applied on the benchmark metabolic pathways, and the results showed that the NCGA achieved better results compared to the existing methods.

## Introduction

Recently, computational system biology has gained attention from many researchers and become an important research area. The computational system biology has contributed to the understanding on the process of the complex biology where it enables the biological process to act as a system. It can be achieved by utilizing the techniques and knowledge in the molecular biology and genetics where it allows living cell to be manipulated as a real factory and gives insights to researchers into ways to improve cell production. One way to improve cell production is by *in silico* optimization of metabolic pathway production. Metabolic pathway can be defined as a series of chemical reactions that occur within the microorganism cell. The computational system biology has enable the metabolic pathway to be represented by mathematical model. Due to that, it is possible to perform the optimization process using computer simulation (*in silico* optimization).

The *in silico* optimization of metabolic pathway production can be seen as the search for a set of components (chemical reactions) for maximizing production rate. It works by altering and tuning the value of the components in the metabolic pathway in order to find the maximum production rate. Similar to the production, the total of the component concentrations involved also needs to be considered. When minimum value of component concentrations are involved, the production cost can be reduced [1, 2]. Recently, many successful works have been published on the *in silico* optimization of metabolic pathway production. Most of these works used continuous optimization approach, such as linear programming method [2–4] and geometric programming method [5, 6]. These approaches usually involve equality and inequality constraints. They also require the definition of the decision variable that requires expert knowledge. Moreover, the search process in the continuous optimization approach totally depends on the decision variable, and this can lead to convergence problem if the decision variable is not accurate [7].

In contrast to the continuous optimization approach, the combinatorial optimization approach works by finding the optimal solution from a finite set of objects. The method functions by using a stochastic operator on a pool of candidate solutions for the optimization problem and makes it more efficient and robust [8]. Due to its stochastic nature, the method does not require any assumption regarding the structure of the model and the definition of the decision variable. This is because the stochastic operator uses random method to determine the search direction, which makes it more robust. For this reason, the present study uses the combinatorial optimization approach for the *in silico* optimization of metabolic pathway production. In this optimization process, metabolic pathway can be viewed as a nonlinear equations system. This is because the metabolic pathway can be described as mathematical model. There are many methods that can be used in dealing with the nonlinear equations system, including numerical, evolutionary and swarm intelligence methods. The Newton method, which is a numerical method, is the most popular method used [9, 10]

Newton method is an iterative method used to find an optimum point to real-valued roots. The utilization of the Newton method for the *in silico* optimization of metabolic pathway production is a good choice because of the fast convergence speed of the Newton method [11]. In this work, Newton method views metabolic pathway as a nonlinear equations system. Applying only the Newton method is not sufficient for the optimization process because the variables in the nonlinear equations system need to be tuned. This is because all the metabolic pathway components are represented by many variables in the nonlinear equations system. Therefore, a method is needed to represent and tune the variables. To overcome this problem, genetic algorithm (GA) is applied. This approach can be performed by representing the variables in the nonlinear equations system as a chromosome. Then, the chromosome is evolved and tuned. However, several issues occur when this method is applied on a complex metabolic pathway that involves many metabolic pathway components. This can cause complex representation of the solution as many metabolic pathway components need to be represented by GA. In addition, evaluating the solution is time consuming. Therefore, a method needs to be embodied into the GA to simplify the representation of the solution. It has been found that applying the cooperative co-evolutionary algorithm (CCA) is the best choice, where the CCA method works by dividing the representation of the solution into multiple sub-solutions [12–14].

In this paper, an improved method, namely the Newton Cooperative Genetic Algorithm (NCGA), was utilized for the *in silico* optimization of metabolic pathway production. This method uses the Newton method to deal with metabolic pathway. GA is then used in the optimization process and CCA is embodied into the GA to decompose the solution into multiple sub-solutions. Furthermore, this study introduced concepts for representing the solution, namely NCGA representation which includes sub-chromosome representation and cooperative chromosome representation. The sub-chromosome is part of the cooperative chromosome, while the cooperative chromosome is a complete solution. The NCGA representation concept ensures that the NCGA is able to increase the production. In addition, this study also introduced a two-level evaluation of the solution, namely sub-chromosome evaluation and cooperative chromosome evaluation where this concept minimizes the total of the component concentrations involved. In the following section, the theory of the metabolic pathway is discussed, where the modelling of the metabolic pathway and the optimization problem are described. This is followed by a discussion of the proposed method and two case studies of the optimization of ethanol production in *Saccharomyces cerevisiae* (*S. cerevisiae*) pathway and the optimization of *tryptophan* (*trp*) biosynthesis in *Escherichia coli* (*E. Coli*) pathway. Finally, the results are presented and discussed before a brief conclusion is made.

## Modeling of metabolic pathway

In this study, the mathematical model used to represent the metabolic pathway is generalized mass action (GMA) models. The representation of GMA has the following form:

$$\begin{array}{c} \frac{dX}{dt}=sv\left(x\right) \end{array}$$ (1)

where *s* represents the stoichiometric matrix of the system and *v*(*x*) denotes the vector that contains reaction rate. There are two types of reactions, which are dependent and independent variables. The dependent variable usually represents the metabolite concentrations, while the independent variable is the enzyme activity. These variables are usually in the form of nonlinear functions. The reaction rate *v*(*x*) can be represented by using the power-law function, which has the following form [6, 15]:

$$\begin{array}{c} v_{i}=\gamma_{i}\prod_{j}x_{j}^{f_{ij}} \end{array}$$ (2)

In this representation, coefficient *γ*_*i*_ is denoted as the rate constant for *v*_*i*_ and coefficient *f*_*ij*_ is the kinetic order. These two coeffcients are derived from the Taylor series in the logarithmic space around a steady state [6, 16]. They can be defined as follows:

$$\begin{array}{c} \gamma_{i}={\left|v_{i}\right|}_{0} \end{array}$$ (3)

$$\begin{array}{c} f_{ij}=|\frac{\delta v_{i}}{\delta x_{j}}\frac{x_{j}}{v_{j}}| \end{array}$$ (4)

where the subscript 0 refers to the value at the steady state condition.

## Problem statement for optimization

The *in silico* optimization of metabolic pathway production is always constrained by steady state condition. In the steady state condition, all the variables in the metabolic pathway are in static values. This condition forces all GMA models to be equal to 0, and thus produces models as follows:

$$\begin{array}{c} \frac{dX_{n}}{dt}=[sv{\left(x\right)}_{1},\;\ldots \;sv{\left(x\right)}_{n}]=0 \end{array}$$ (5)

This leads to the solving of a nonlinear equations system, which can be defined as follows [1, 9, 10, 17]:

$$\begin{array}{c} f(x)=[f{\left(x\right)}_{1},f{\left(x\right)}_{2},\ldots ,f{\left(x\right)}_{n}]=0 \end{array}$$ (6)

where *x* = (*x*_1_, *x*_2_⋯*x*_*n*_) denotes *n* equations and *n* variables, while *f*_1_, *f*_2_⋯*f*_*n*_ are the nonlinear functions. In order to solve a nonlinear equations system, all the equations *f*(*x*)_1_, *f*(*x*)_2_⋯*f*(*x*)_*n*_ must be equal to 0. This situation is similar to the *in silico* optimization of metabolic pathway production. Therefore, the *in silico* optimization of metabolic pathway production can be considered as a method for solving a nonlinear equations system.

Besides the steady state condition, the constraint of the components in the metabolic pathway also needs to be considered. This constraint exists in order to ensure the concentrations of the components remain within the boundaries to maintain the survival of the microorganism cell [1, 6]. Thus, the *in silico* optimization of metabolic pathway production involves improving the pathway production and at the same time tries to reduce the component concentration involved. The optimization problem can be written as follows:

$$\begin{array}{c} max\;F_{1}\;(v) \end{array}$$ (7)

$$\begin{array}{c} min\;F_{2}\;(\sum_{j=1}^{n}x_{j})\; \end{array}$$ (8)

where objective function *F*_1_ is the production (maximum function) and objective function, while *F*_2_ is the total of the component concentrations involved.

## Newton cooperative genetic algorithm

In this section, NCGA method is discussed. The NCGA is used for the *in silico* optimization of metabolic pathway production. The proposed method treats the metabolic pathway as a nonlinear equations system. In dealing with a nonlinear equations system, the Newton method is applied, and then GA is used to represent the variables in the system. This is necessary in order to tune the variables to search for the optimum set. GA loses its effectiveness when it is applied in a complex metabolic pathway as the representation of the solution becomes complex and difficult to evaluate. Hence, CCA is applied to overcome this particular problem.

**Fig 1:**
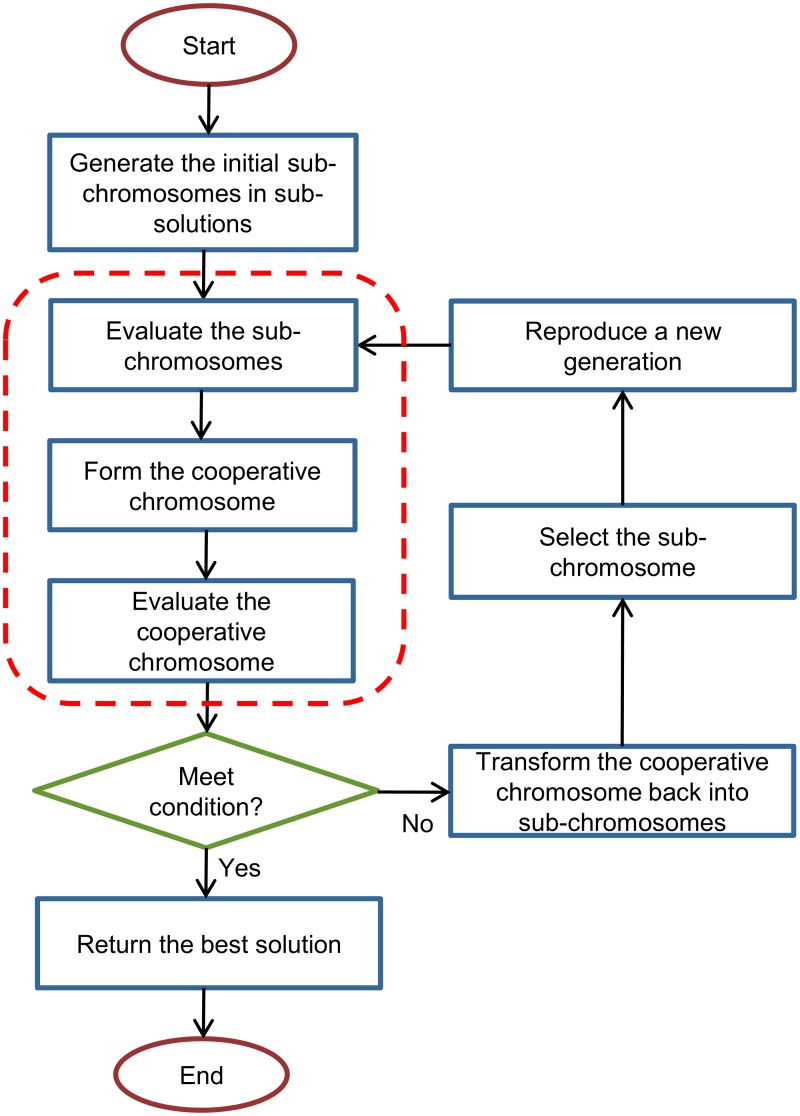
The flow chart of the NCGA. The NCGA starts with representation of the solution by GA and CCA. Then the Newton method is applied in order to evaluate the solution.

**Fig 2:**
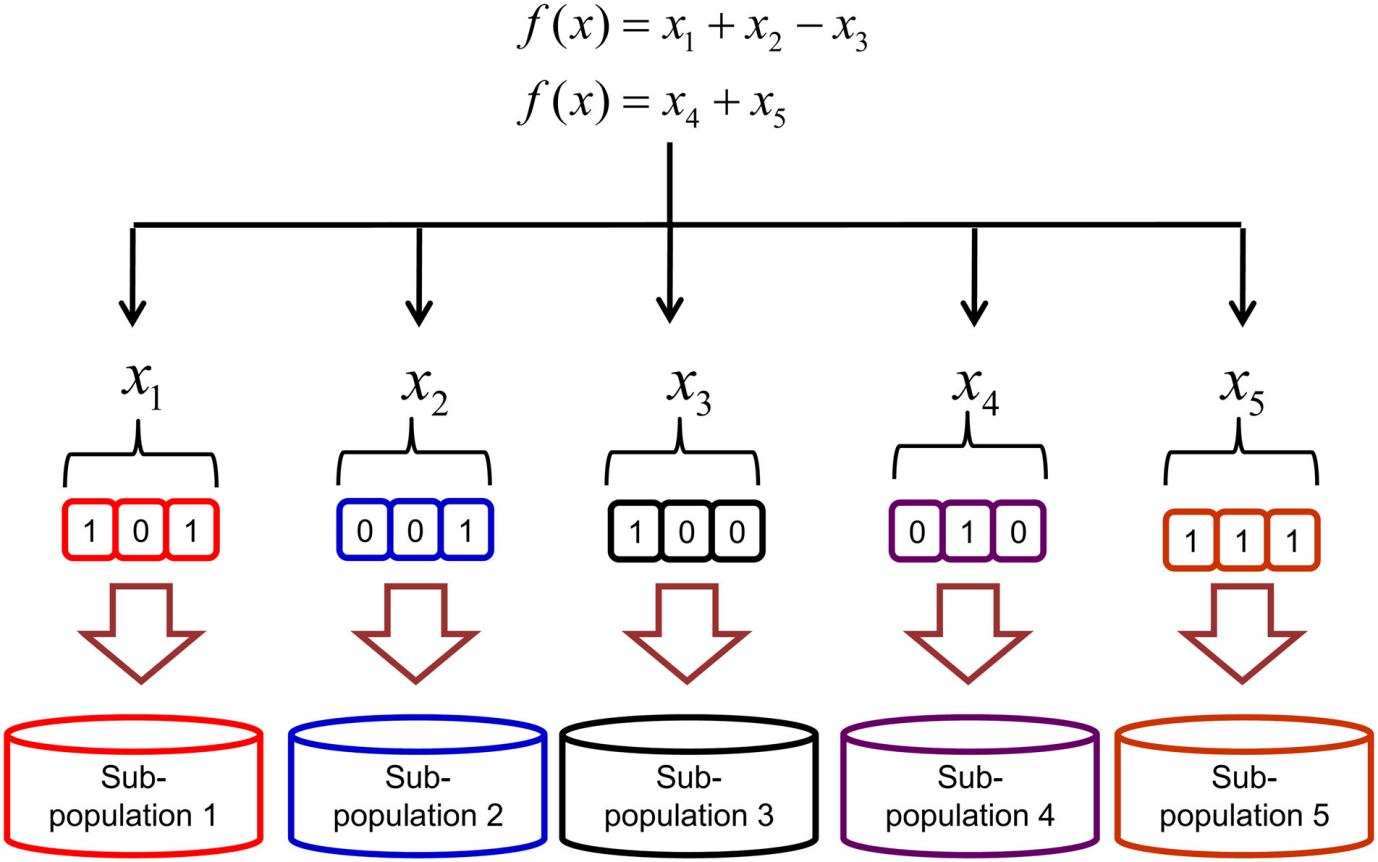
The representation of nonlinear equations system variables by sub-chromosomes. Each variable in nonlinear equations system represented by sub-chromosome and evolve in their own sub-population.

The present study introduces a modified GA, which includes the NGCA representation concepts and the two-level fitness evaluation. Fig 1 shows the simplified flow chart of the NCGA. In the figure, the concepts of the NCGA representation and the two-level fitness evaluation are highlighted by the dashed lines. The detailed flow of the algorithm is as follows:

Step 1. Generate the initial *N* sub-chromosome in *N* sub-population. Each sub-chromosome represents each variable in the nonlinear equations system. The number of *N* sub-populations depends on the number of variables in the nonlinear equations system. The example of representation of all variables in nonlinear equations system by sub-chromosomes can be viewed in Fig 2. The sub-chromosome is in binary representation.

Step 2. Evaluate the sub-chromosomes. In this step, a representative of the sub-chromosome from all the sub-populations is selected based on the fitness value, where the sub-solution with the lowest fitness value is selected first. This is done to ensure that the representatives from all the sub-populations will combine with each other in order to minimize the total component concentrations involved. This step is referred to as the sub-chromosome evaluation.

**Fig 3:**
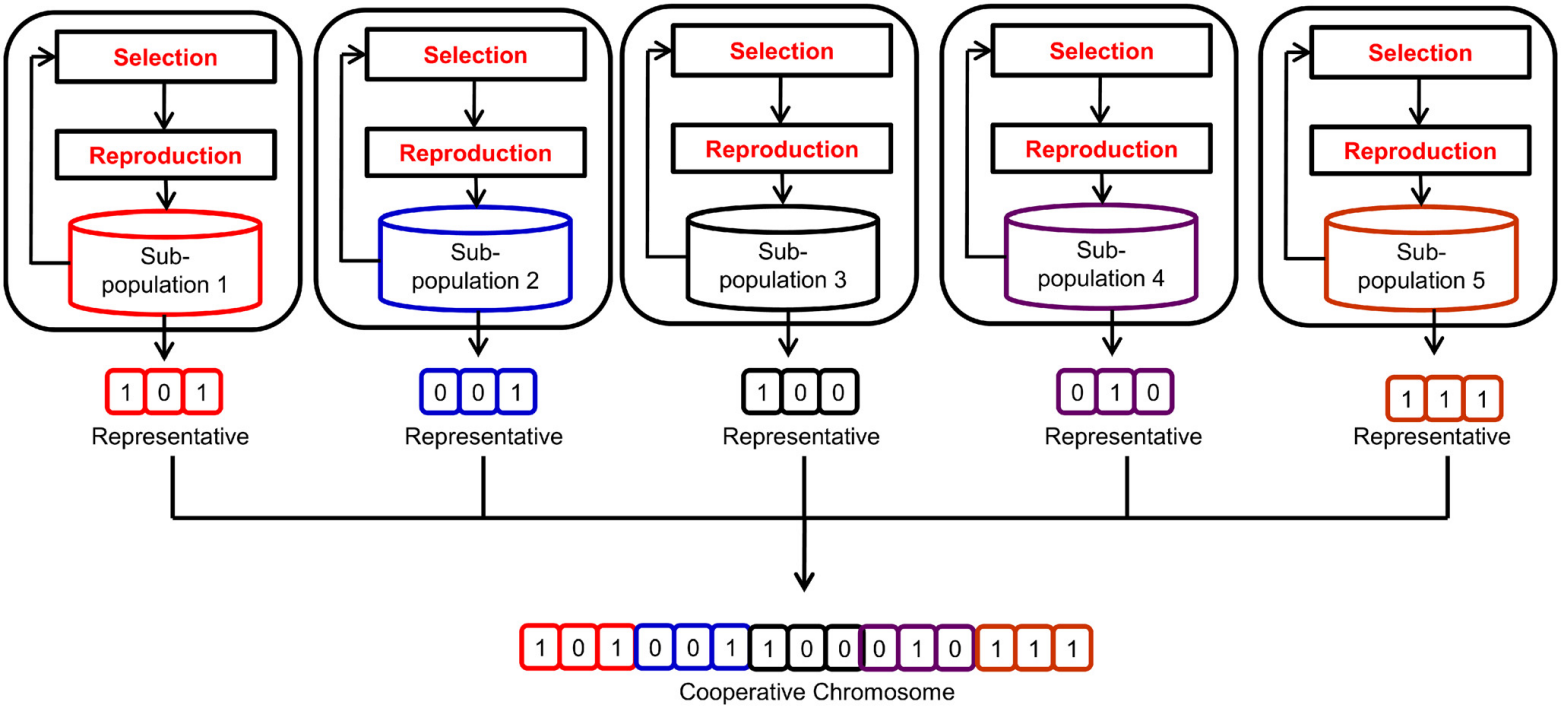
Cooperative chromosome representation. A representative was selected from all sub-population in order to form the cooperative chromosome.

Step 3. Form the cooperative chromosome. After all the representatives are selected from their sub-populations, they are combined with each other to form a complete solution known as the cooperative chromosome. This concept, referred to as the cooperative chromosome representation, is pictured in Fig 3.

Step 4. Evaluate the cooperative chromosome. In this step, the cooperative chromosome is tested by the Newton method. A condition at this stage is whether or not termination has occurred. The termination condition can occur in two conditions; when the maximum number of generations has been reached, and when the component concentration constraint is in the optimum range. If the termination condition is achieved, then the process skips directly to Step 8.

Step 5. Transform the cooperative chromosome back into sub-chromosomes. In this step, the cooperative chromosome is decomposed back into sub-solutions with all the sub-chromosomes are going back into their own sub-population. The purpose of this step is to select sub- chromosomes in order to perform the reproduction process.

Step 6. Select the sub-chromosome. The selection process is based on the fitness values of the sub-chromosome, whereby the sub-chromosome with the lowest fitness value is selected first. This is intended to minimize the total of the component concentrations involved by making all sub-solutions that have lowest fitness value combine with each other. The process is performed until the last sub-chromosome has been selected.

Step 7. Reproduce a new generation. Crossover and mutation procedures are applied on the selected sub-chromosomes. This is performed in order to produce a new generation that has better quality compared to the previous generation. Then, the new generation goes back to Step 2.

**Fig 4:**
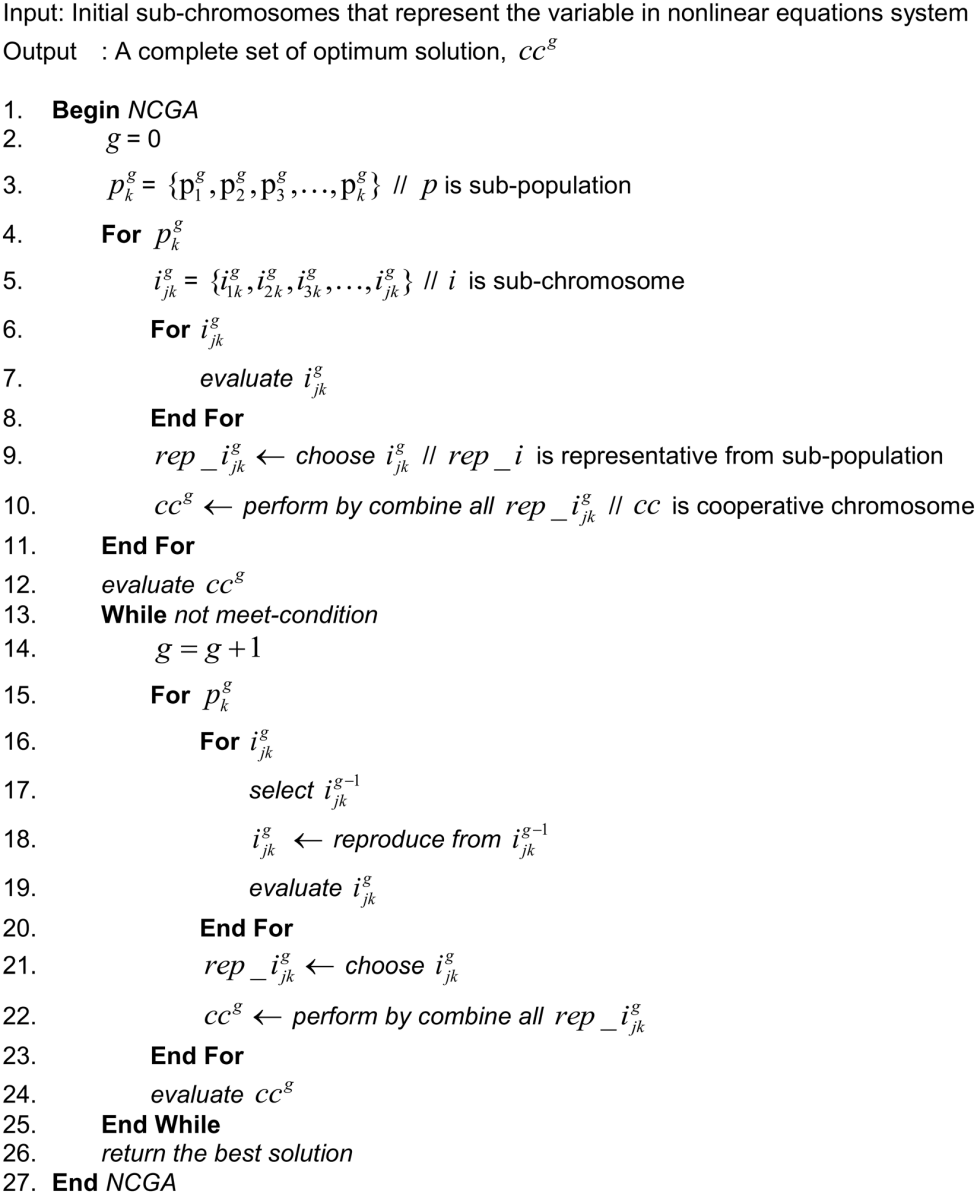
The pseudocode of the Newton cooperative genetic algorithm.

Step 8. Return the best solution. This step decodes the cooperative chromosome into the solution. Fig 4 shows the NCGA algorithm in pseudocode format.

## Case studies

To prove the effectiveness of the NCGA, a program was used and tested on the metabolic pathway benchmark, namely the optimization of ethanol production in *S. cerevisiae* and the optimization of *trp* production in *E. coli* pathway. The program written in Java language that is known as jMetal [18] was used. Besides that, JAMA version 1.0.3 [19] was used to handle the nonlinear equations system and integrate with the jMetal program. The jMetal program can be downloaded from http://jmetal.sourceforge.net/index.html while JAMA version 1.0.3 from http://math.nist.gov/javanumerics/jama/.

### Case study 1: Optimization of ethanol production in *S. cerevisiae* pathway

#### Modelling framework

**Fig 5:**
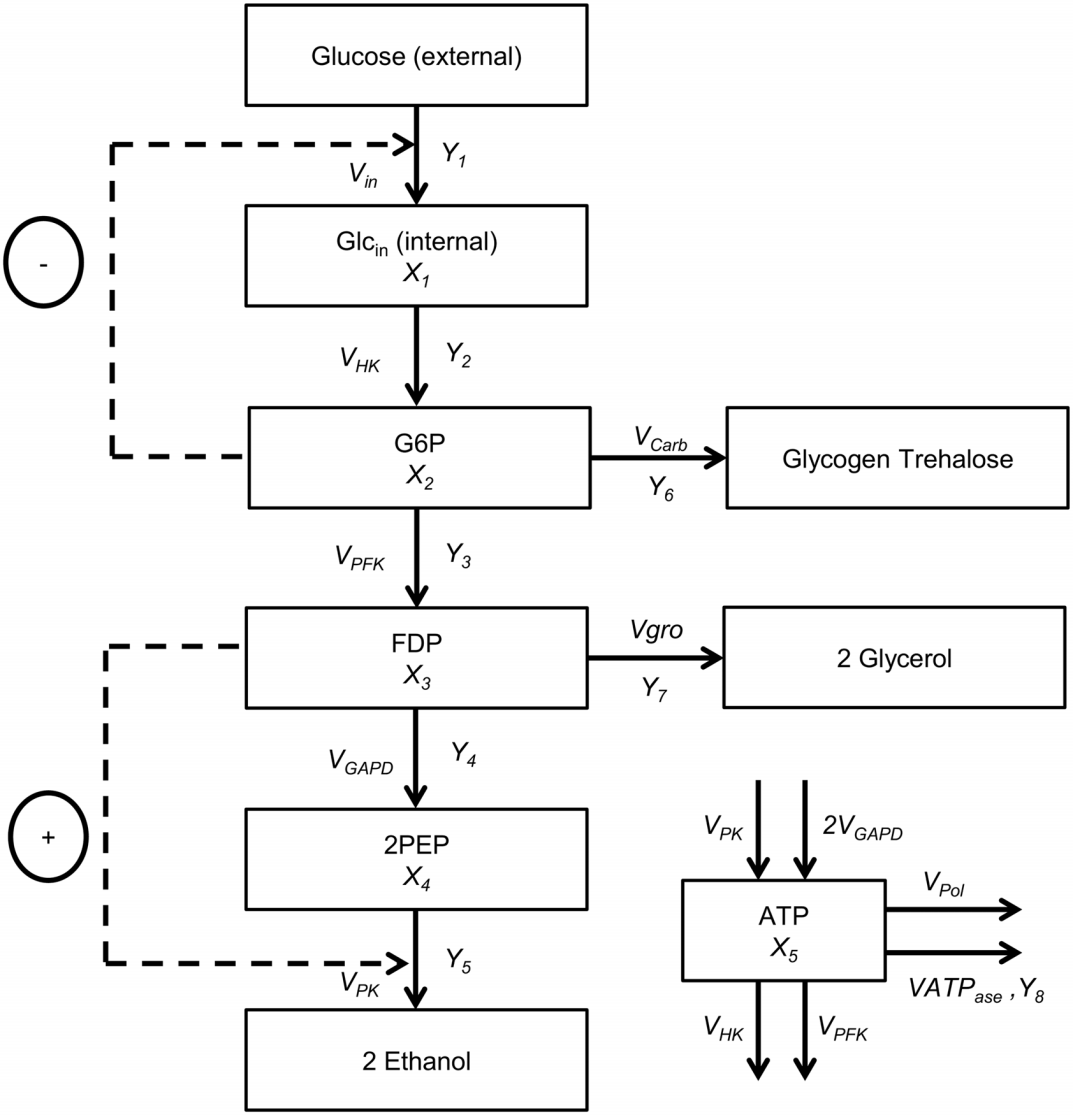
Anaerobic fermentation pathway in *S. cerevisiae*.

The GMA model derived from the ethanol production by *S. cerevisiae* was used as the case study in order to observe the performance of NCGA. The GMA model derived from the *S. cerevisiae* pathway was suspended in a cell culture at pH 4.5 as recommended by Galazzo and Bailey [20]. This pathway has been applied in many studies over the years. Fig 5 depicts a schematic representation of the *S. cerevisiae* pathway. The GMA model of this pathway is described as follows:

$$\begin{array}{ccc} \frac{dX_{1}}{dt} & = & V_{in}-V_{HK}\\ \frac{dX_{2}}{dt} & = & V_{HK}-V_{PFK}-V_{Carb}\\ \frac{dX_{3}}{dt} & = & V_{PFK}-V_{GAPD}-0.5V_{Gro}\\ \frac{dX_{4}}{dt} & = & 2V_{GAPD}-V_{PK}\\ \frac{dX_{5}}{dt} & = & 2V_{GAPD}+V_{PK}-V_{HK}-V_{Carb}-V_{PFK}-V_{ATPase} \end{array}$$ (9)

The variables and the corresponding steady state are summarized in Table 1. At the initial steady state, all the fluxes in the model were formulated in the following form:

$$\begin{array}{ccc} & & V_{in}=0.8122X_{2}^{-0.2344}Y_{1}\\ & & V_{HK}=2.8632X_{1}^{0.7464}X_{5}^{0.0243}Y_{2}\\ & & V_{PFK}=0.5232X_{2}^{0.7318}X_{5}^{-0.3941}Y_{3}\\ & & V_{Carb}=8.904\times 10^{-4}X_{2}^{8.6107}Y_{7}\\ & & V_{GAPD}=7.6092\times 10^{-2}X_{3}^{0.6159}X_{5}^{0.1308}Y_{4}\\ & & V_{Gro}=9.272\times 10^{-2}X_{3}^{0.05}X_{4}^{0.533}X_{5}^{-0.0822}Y_{8}\\ & & V_{PK}=9.471\times 10^{-2}X_{3}^{0.05}X_{4}^{0.533}X_{5}^{-0.0822}Y_{5}\\ & & V_{ATPase}=X_{5}Y_{6} \end{array}$$ (10)

**Table 1:** Summary of metabolites and enzymes in case study 1.

**Metabolite**	**Acronym**	**Symbol**	**Initial Steady State**
Glucose (internal)	*Glc*_*in*_	*X*_1_	0.0345
Glucose-6-phosphate	*G*6*P*	*X*_2_	1.0110
Fructose-1,6-phosphate	*FDP*	*X*_3_	9.1440
Phosphoenolpyruvate	*PEP*	*X*_4_	0.0095
Adenosine triphosphate	*ATP*	*X*_5_	1.1278
**Enzyme**	**Acronym**	**Symbol**	**Initial Steady State**
Glucose transport	*V*_*in*_	*Y*_1_	19.70
Hexokinase	*V*_*HK*_	*Y*_2_	68.50
Phosphofructo-1-kinase	*V*_*PFK*_	*Y*_3_	31.70
Glyceraldehyde dehydrogenase	*V*_*GAPD*_	*Y*_4_	49.90
Pyruvate kinase	*V*_*PK*_	*Y*_5_	3440.00
ATPase	*V*_*ATPase*_	*Y*_8_	25.10
Polysaccharide biosynthesis	*V*_*Carb*_	*Y*_6_	14.31
Polyol biosynthesis	*V*_*Gro*_	*Y*_7_	203.00

#### Optimization problem

The performance of the method proposed in this work can be assessed by the rate of ethanol production given by the flux of pyruvate kinase, *V*_*PK*_. In addition, the total of the component concentrations involved must be considered. Thus, the optimization problem of this case study can be defined as follows:

$$\begin{array}{c} max\;F_{1}\;(v)=V_{PK} \end{array}$$ (11)

$$\begin{array}{c} min\;F_{2}=\sum_{j=1}^{5}X_{j}+\sum_{j=6}^{6}Y_{j} \end{array}$$ (12)

The optimization problem was subjected to the steady state condition, where all the GMA models were equal to 0:

$$\begin{array}{ccc} & & V_{in}-V_{HK}=\;\;0\\ & & V_{HK}-V_{PFK}-V_{Carb}=\;\;0\\ & & V_{PFK}-V_{GAPD}-0.5V_{Gro}=\;\;0\\ & & 2V_{GAPD}-V_{PK}=\;\;0\\ & & 2V_{GAPD}+V_{PK}-V_{HK}-V_{Carb}-V_{PFK}-V_{ATPase}=\;\;0 \end{array}$$ (13)

The constraint of the component concentrations involved was also considered. This is to ensure that the microorganism is still workable. In this case study, the components were categorized into two groups, which are metabolite and enzyme. The metabolites (*X*_1_ − *X*_5_) were set approximately 20% from their steady state values, which were in the range of 0.8–1.2. For the enzymes, not all enzymes were tuned as only *Y*_1_ − *Y*_6_ were involved. The values for enzymes were set in the range of 0–50 [1, 21].

### Case study 2: Optimization of *trp* biosynthesis in *E. Coli* pathway

#### Modelling framework

**Fig 6:**
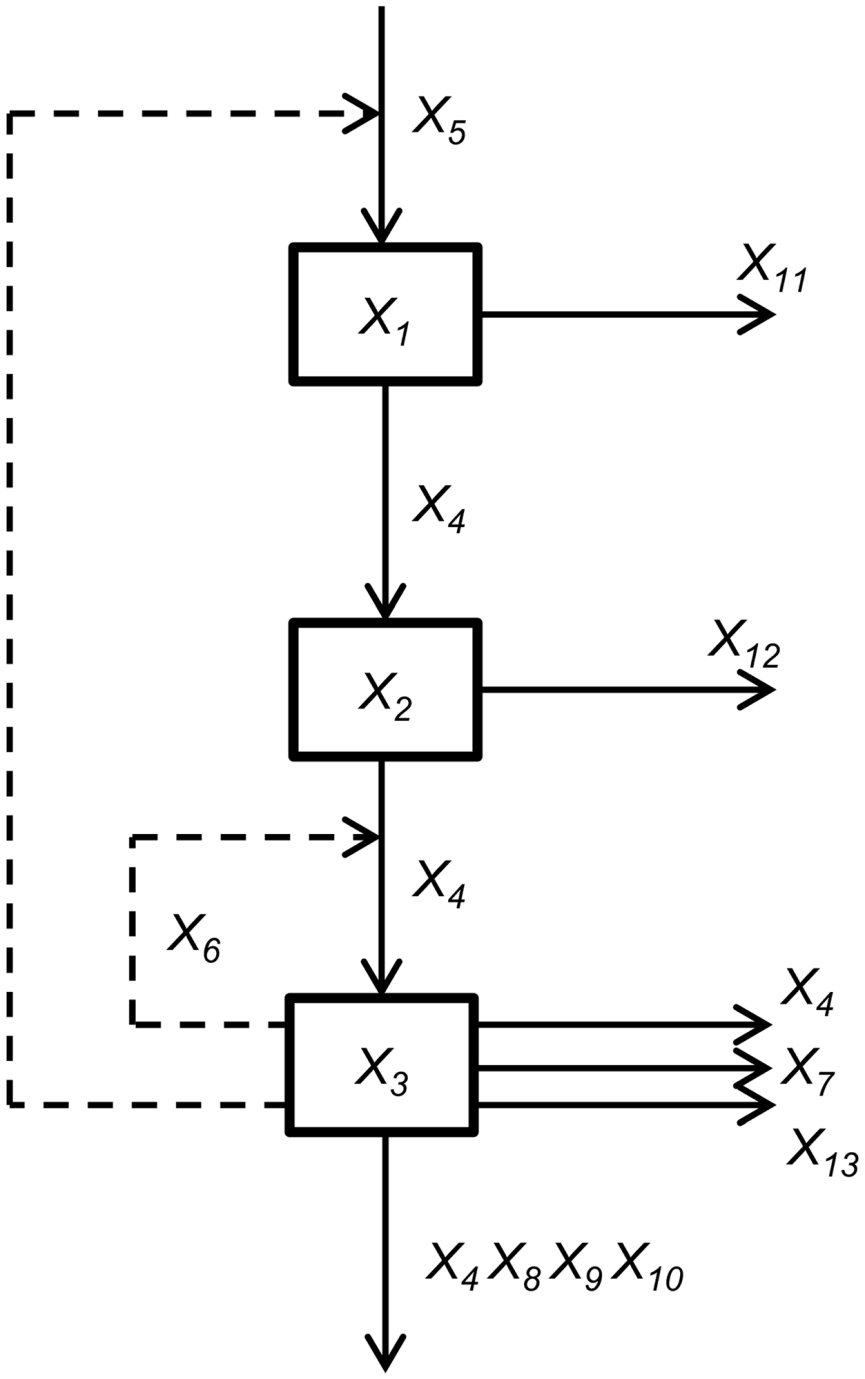
*Tryptophan* biosynthesis in *E. coli* pathway.

In this pathway, NCGA was used to optimize the *trp* production. This pathway is introduced by Xiu *et al*. and detailed description can be found in [22]. This pathway is depicted in Fig 6 and the detail is given in Table 2. This pathway has the following GMA model:

$$\begin{array}{ccc} \frac{dX_{1}}{dt} & = & V_{11}-V_{12}\\ \frac{dX_{2}}{dt} & = & V_{21}-V_{22}\\ \frac{dX_{3}}{dt} & = & V_{31}-V_{32}-V_{33}-V_{34} \end{array}$$ (14)

where *X*_1_ is the mRNA concentration, *X*_2_ is the enzyme concentration and *X*_3_ is the *trp* concentration. In steady state condition, all components in this pathway (variables in Table 2) have the following rate:

$$\begin{array}{ccc} V_{11} & = & 0.6403X_{3}^{-5.87\times 10^{-4}}X_{5}^{-0.8332}\\ V_{12} & = & 1.0233X_{1}X_{4}^{0.0035}X_{11}^{0.9965}\\ V_{21} & = & X_{1}\\ V_{22} & = & 1.4854X_{2}X_{4}^{-0.1349}X_{12}^{0.8651}\\ V_{31} & = & 0.5534X_{2}X_{3}^{-0.5573}X_{6}^{0.5573}\\ V_{32} & = & X_{3}X_{4}\\ V_{33} & = & 0.9942X_{3}^{7.0426\times 10^{-4}}X_{7}\\ V_{34} & = & 0.8925X_{3}^{3.5\times 10^{-6}}X_{4}^{0.9760}X_{8}X_{9}^{-0.0240}X_{10}^{-3.5\times 10^{-6}} \end{array}$$ (15)

**Table 2:** Details of component concentrations in case study 2.

**Variable**	**Initial Steady State**
*X*_1_	0.184654
*X*_2_	7.986756
*X*_3_	1418.931944
*X*_4_	0.00312
*X*_5_	5
*X*_6_	2283
*X*_7_	0.011
*X*_8_	430
*X*_9_	7.5
*X*_10_	0.005
*X*_11_	0.9
*X*_12_	0.02
*X*_13_	0

#### Optimization problem

The first objective function was to maximize the *trp* production given by the reaction of *V*_34_ [23]. In addition, the second objective function was to minimize the total concentrations involved. Thus, the optimization problem of this case study can be defined as follows:

$$\begin{array}{c} max\;F_{1}\;=V_{34} \end{array}$$ (16)

$$\begin{array}{c} min\;F_{2}\;=\sum_{j=1}^{6}X_{j}+X_{8} \end{array}$$ (17)

Similar to case study 1, the optimization problem was subjected to the steady state condition. Hence, this produced GMA models with the following form:

$$\begin{array}{ccc} & & V_{11}-V_{12}=0\\ & & V_{21}-V_{22}=0\\ & & V_{31}-V_{32}-V_{33}-V_{34}=0 \end{array}$$ (18)

In this case study, not all of the components were tuned. Only seven components were tuned, which are *X*_1_ − *X*_6_ and *X*_8_. For *X*_1_ − *X*_3_, the component concentration constraints were in the range of 0.8–1.2; for *X*_4_, the component concentration constraints were in the range of 0–0.00624; for *X*_5_, the component concentration constraints were in the range of 5–10; for *X*_6_, the component concentration constraints were in the range of 500–5000 and for *X*_8_, the component concentration constraints were in the range of 0–1000 [4–6].

## Results and discussion

Results and discussion Many parameter settings were used while performing the experiments. Table 3 summarizes the parameter settings used when the best result was obtained. For the Newton method, fixed parameters were used; the maximum number of iterations was fixed to 50 and the tolerance was set to 10^−6^.

**Table 3:** Summary of parameter settings in producing the best result.

**Parameter**	**Case Study 1**	**Case Study 2**
Number of sub-populations	11	7
Number of individuals in sub-populations	150	150
Maximum generation	300	350
Crossover point	2.0	1.0
Mutation rate	0.3	0.2

In case study 1, the full results obtained by the NCGA are presented in Table 4. The table gave the best result achieved, the average of 100 experiments, the standard deviation and the comparison with other works. For the best ethanol production, the NCGA was able to increase the ethanol production up to 52.91 times from its initial steady state value. Several other works were compared with the NCGA in order to assess its performance. As seen in the table, the NCGA produced the highest amount of ethanol compared to the others. Besides that, the NCGA was able to reduce the total amount of the component concentrations involved, with the total concentration was only 294.80.

**Table 4:** Best solution obtained using the NCGA in case study 1.

**Variable**	**Best Solution**	**Average of 100 experiments**	**Standard deviation**	**Rodriguez-Acosta *et al*. [21]**	**Xu [6]**	**Ismail *et al*. [24]**
*X*_1_	1.1121	0.9940	0.1112	1.14	1.102	1.11
*X*_2_	1.0301	1.0000	0.1136	1.05	1.046	1.03
*X*_3_	1.1874	1.0057	0.1281	1.15	1.141	1.13
*X*_4_	1.1707	1.1273	0.0392	1.17	1.171	1.18
*X*_5_	0.9116	0.9839	0.1129	1.12	1.113	1.14
*Y*_1_	49.73	49.9811	0.0271	49.97	50	49.99
*Y*_2_	45.8097	45.0758	0.1806	44.77	45.953	45.83
*Y*_3_	48.8933	49.9056	0.1039	49.89	50	49.92
*Y*_4_	48.13	47.3806	0.1273	47.26	47.772	47.97
*Y*_5_	47.85	49.3487	0.5269	48	48.366	48.30
*Y*_6_	48.9782	49.7864	0.1262	49.75	50	49.79
*F*_1_	52.91	52.74	0.0133	52.0843	52.5118	52.57
*F*_2_	294.80	295.19	0.3274	295.27	297.664	297.384

The complete result achieved using NCGA in case study 2 is given by Table 5. The table summarizes the best result achieved, the average of 100 experiments, the standard deviation and the comparison with other works. For the best solution, NCGA was able to produce *trp* up to 3.9759 times the initial steady state value. When the comparison was made with other works, the result produced by NCGA was the highest. For the total component concentrations involved, NCGA was able to reduce the total amount of the component concentrations involved to 6006.5581, where it was the lowest value compared to other works.

**Table 5:** Best solution obtained using the NCGA in case study 2.

**Variable**	**Best Solution**	**Average of 100 experiments**	**Standard deviation**	**Marin-Sanguino *et al*. [5]**	**Vera *et al*. [4]**	Xu [6]	**Ismail *et al*. [24]**
*X*_1_	0.9053	1.0741	0.0995	1.1900	1.2000	1.2000	1.1100
*X*_2_	0.8302	1.1091	0.0794	1.1480	1.1500	1.1150	1.1140
*X*_3_	0.8000	0.8000	1.56*e* ^−15^	0.8000	0.8000	0.8000	0.8000
*X*_4_	0.0054	0.0054	1.14*e* ^−5^	000041	0.0040	0.0054	0.00538
*X*_5_	4.0172	4.4736	0.3071	4.0000	4.0000	4.0110	4.7540
*X*_6_	5000	5000	0	5000	5000	5000	5000
*X*_8_	1000	1000	0	1000	1000	1000	1000
*F*_1_	3.9759	3.9614	0.0032	3.0620	3.0620	3.9460	3.9570
*F*_2_	6006.5581	6006.9621	0.4075	6007.1412	6007.1540	6007.1314	6007.7814

**Fig 7:**
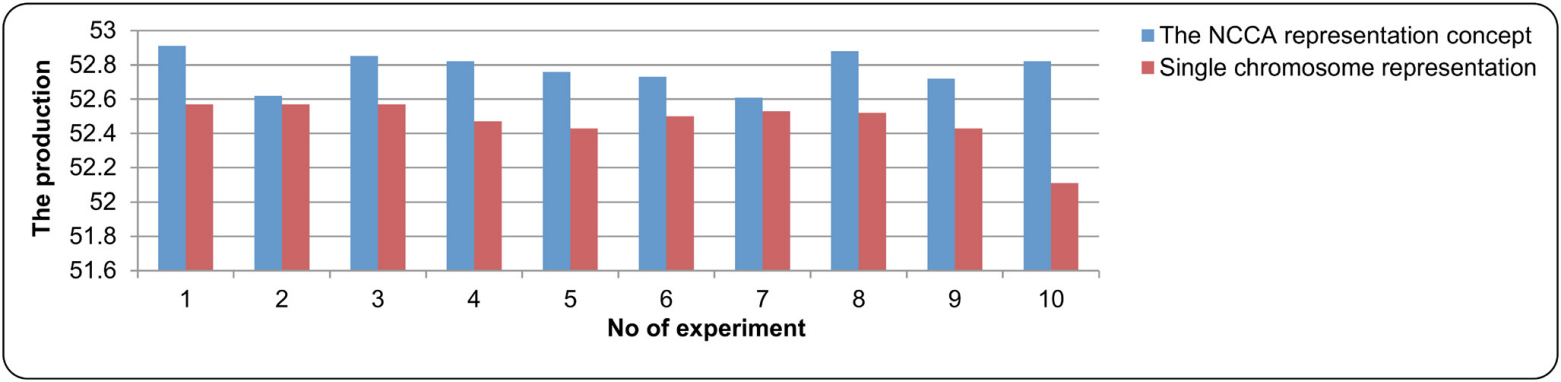
The comparison results of the NCGA representation concept in case study 1.

**Fig 8:**
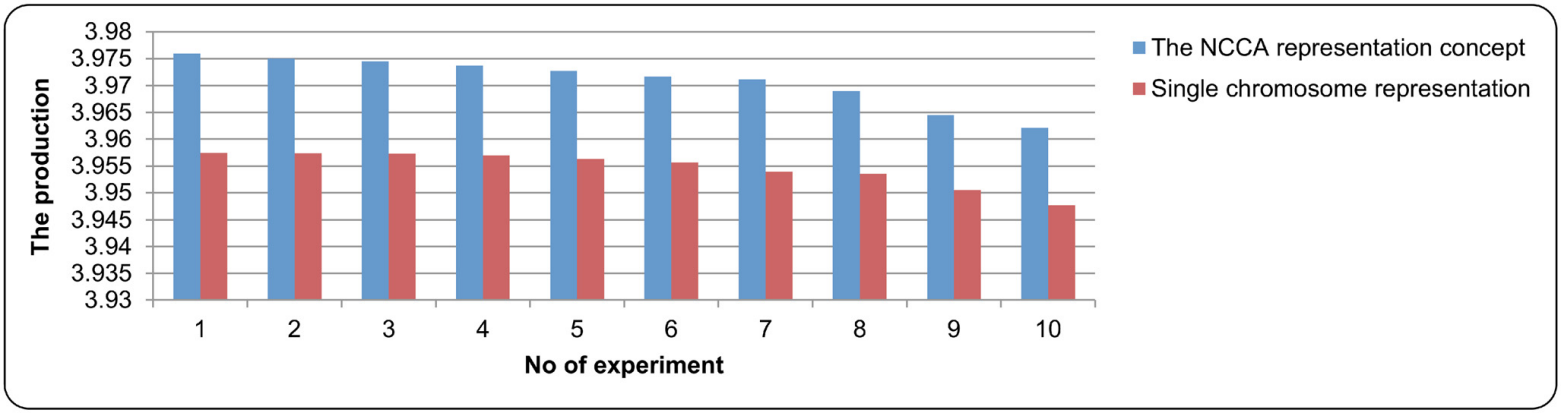
The comparison results of the NCGA representation concept in case study 2.

In evaluating the concept of the NCGA representation, it was compared with the single chromosome representation (traditional GA). Several experiments were conducted in evaluating this concept and the parameter settings as indicated in Table 3 were used. Fig 7 and Fig 8 give the comparison of the results. From both figures, it can be observed that all the production results that utilized the NCGA representation concept were higher compared to the results that only used single chromosome representation. This is because each variable in a nonlinear equations system was represented by multiple sub-chromosomes. In contrast to that, a single representation of chromosome represents all the variables of nonlinear equations system into a single chromosome. As the concept of NCGA representation allowed each variable in the nonlinear equations system to be represented by many sub-chromosomes and reproduced in their own sub-population, this made all variables were tuned in order to produce the optimum result. This did not happen in the single representation of the solution, as there were possibilities that not all variables were tuned because all variables were grouped together into only a single representation. As a conclusion, applying the concept of sub-chromosome and cooperative chromosome representation enables the improvement of the production.

**Fig 9:**
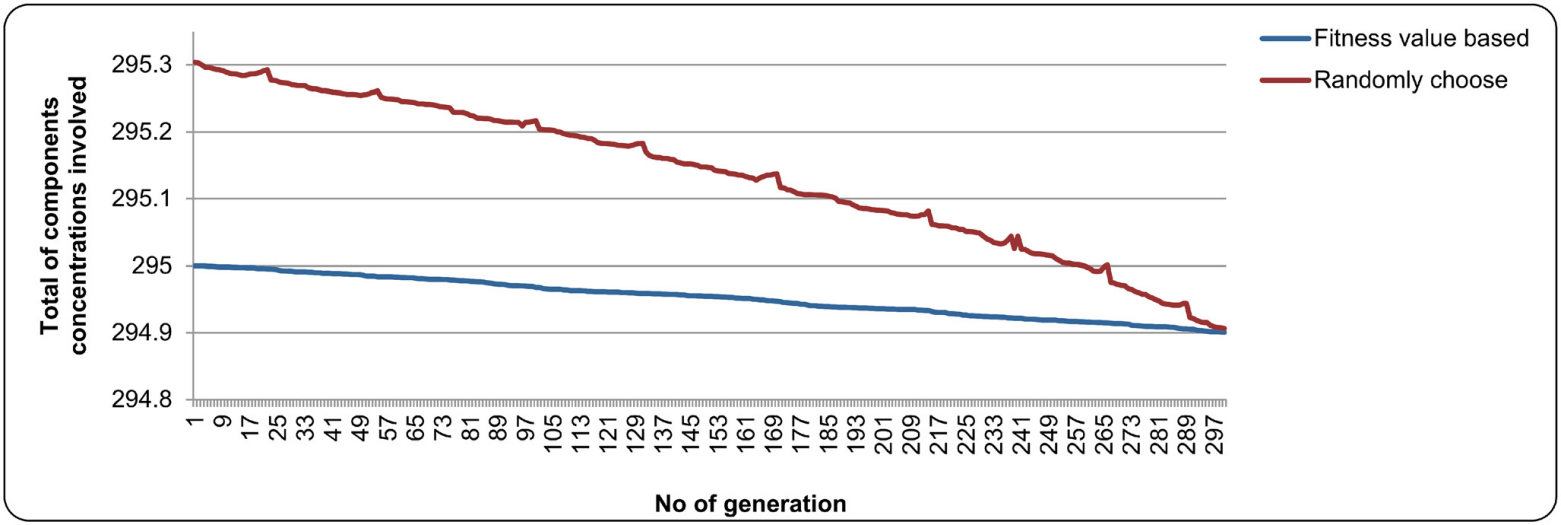
The result of two-fitness evaluation concept in case study 1.

**Fig 10:**
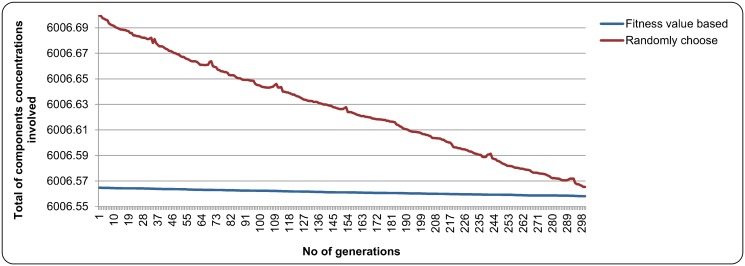
The result of two-fitness evaluation concept in case study 2.

In validating the two-level evaluation concept that was introduced in this study, several experiments were conducted where the representatives from all sub-populations were selected randomly based on their fitness value. The experiments used the parameter settings in Table 3 and the number of maximum generation was fixed to 300. The graph in Fig 9 and Fig 10 depict the results obtained in case study 1 and case study 2 respectively. In both figures, it was found that the representatives selected based on their fitness value were able to minimize the total of the component concentrations involved compared to the representatives that were selected randomly. Furthermore, the total of the component concentrations involved for the representatives that were selected based on the fitness value in Fig 9 and Fig 10 decreased slightly from the 1^*st*^ generation to the 300^*th*^ generation. For the total of the component concentrations involved of the randomly selected representatives, it was found that the total of component concentrations involved in the next generation sometimes were higher than the previous generation. This might be due to the random element in selecting the representatives to form cooperative chromosome. This could happen when a sub-chromosome in the current generation with higher fitness value compared to others in their sub-population was selected randomly as a representative, and then combined with other representatives from other sub-populations. This made the total of the component concentrations involved became higher in the next generation. In conclusion, the two-level evaluation concept that was introduced in this study was able to reduce the total of components concentrations involved and allow the NCGA to perform the optimization process smoothly.

Several experiments were carried out in order to investigate the reliability of the results obtained by the NCGA. The reliability of the results can be assessed by the average results that are given in Table 4 for case study 1 and Table 5 for case study 2. For case study 1, it could be observed that all component concentrations involved were in their optimum range, thus proved that the NCGA was able to produce reliable results. In addition, the ethanol production rate was higher and the total of the component concentrations involved was the lowest compared to the previous works. Based on this observation, it can be concluded that the NCGA demonstrated the reliability in handling the optimization problem in case study 1. In case study 2, it was found that all component concentrations involved were placed in their optimal range, therefore confirmed that the NCGA was capable of producing reliable result. Besides that, the *trp* production rate was higher and the NCGA was able to reduce more for the total of the component concentrations involved compared to previous works. Based on this finding, the NCGA has shown its ability in the optimization of case study 2.

Besides comparing with previous works, the production of NCGA was also being compared with the Newton method with traditional GA (single chromosome representation). 100 experiments were performed and the best result, the average and the standard deviation were recorded. For the results that were produced by NCGA in case study 1 and case study 2 are given in Table 4 and Table 5 5 respectively, while the result that was produced by the single chromosome representation is as follows; for case study 1, the best solution result for the ethanol production rate is 52.57 and the total of the component concentrations involved is 297.38, the average for the ethanol production rate is 52.40 and the total of the component concentrations involved is 296.65, and the standard deviation for the ethanol production rate is 0.0826 and the total of the component concentrations involved is 0.3944; for case study 2, the best result for the *trp* production rate is 3.9570 and the total of the component concentrations involved is 6007.7884, the average for the *trp* production rate is 3.9510 and the total of the component concentrations involved is 6007.9811, and the standard deviation for the *trp* production rate is 0.0049 and the total of the component concentrations involved is 0.4872. It can be observed that all the production result produced by NCGA was higher compared to the result that was produced by traditional GA in terms of the best solution and the average for all case studies. For the total of the component concentrations involved results (the best solution and the average), NCGA was able to reduce more compared to the Newton method with traditional GA. For the standard deviation, NCGA was able to achieve smaller value of standard deviation compared to the Newton method with traditional GA. This shows that the NCGA is able to find the similar result. This is because with smaller value of the standard deviation points out the consistency and precision of the NCGA.

Other than the production and the total of the component concentrations involved, the computation time (in second) in performing experiment also needs to take into account. Table 6 gives the comparison of the computation time taken in performing experiment for NCGA and Newton method with traditional GA. Generally, the result shows that the NCGA requires more computation time compared to the Newton method with traditional GA. This might be due to the concept of the NCGA representation and the two-level evaluation concept where NCGA needs more time to perform these two processes in order to obtain good results compared to the Newton method with traditional GA.

**Table 6:** The comparison of computation time.

**Method**	**Case study 1 (s)**	**Case study 2 (s)**
NCGA	80.40	40.45
Newton method with traditional GA	80.25	40.31

## Conclusion

In this paper, an improved method for *in silico* optimization of metabolic pathway production known as NCGA has been proposed. The NCGA was developed to improve the metabolic pathway production and at the same time minimize the total concentration of the components involved. The NCGA comprises a combination of Newton method, GA and CCA. The Newton method deals with the metabolic pathway and the GA is employed in the optimization process, with the GA representing the components in the metabolic pathway as a solution. However, as explained in this paper, the representation of the solution becomes complex when it is applied in a complex metabolic pathway. To overcome this situation, the use of the CCA was proposed in order to simplify the representation of the solution by decomposing the solution into multiple sub-solutions. In addition, this study introduced two concepts to make the NCGA perform well, namely the concept of representing the solution and a two-level evaluation of the solution. The proposed method was applied in two case studies, and it showed better results than those reported in previous studies.
